# Giant faecaloma—a rare cause of life-threatening lower gastrointestinal haemorrhage

**DOI:** 10.1259/bjrcr.20150227

**Published:** 2015-10-12

**Authors:** Flavius Parvulescu, Tze Yuan Chan, Ufuk Gur, Richard Gregory McWilliams

**Affiliations:** ^1^ Department of Radiology, Royal Liverpool University Hospital, Liverpool, UK; ^2^ Department of Colorectal Surgery, Royal Liverpool University Hospital, Liverpool, UK

## Abstract

Chronic constipation and faecal impaction are common in the elderly, particularly in institutionalized patients and those with neurological impairment. Faecaloma formation is an extreme manifestation of coprostasis that can lead to stercoral ulcerations and perforation, a recognized severe complication. We present the case of an uncommon life-threatening complication resulting from a giant rectal faecaloma, which has rarely been reported in the literature. The patient presented with haemodynamic shock from profuse per-rectum haemorrhage. Clinical examination revealed a hard central abdominal mass and triple-phase CT of the abdomen demonstrated a tumour-like mass of hard stool in the rectum measuring up to 25 cm and stretching the adjacent vasculature, causing intraluminal active arterial haemorrhage. Emergency selective arterial embolization performed by the interventional radiologists successfully controlled the bleeding with a good outcome. This case highlights a rare but possibly fatal complication of chronic constipation and emphasizes the importance of having access to an acute interventional radiology service capable of promptly dealingwith life-threatening presentations.

## Introduction

Faecalomas are tumour-like masses of dry, hard stool, virtually always located in the rectum or sigmoid colon and associated with a history of chronic constipation. Although there are a few reports in the literature of giant faecalomas in young patients and children,^1^^–^^3^ they are typically seen in elderly patients, particularly those bed bound and institutionalized, in whom faecal impaction is considered the most frequent cause of large bowel obstruction.^4,5^ Complications of this extreme form of coprostasis may vary from subacute colonic obstruction to pressure symptoms caused by mass effect, such as hydronephrosis or urinary frequency, to stercoral colitis, which can lead to perforation and fatal peritonitis. The latter is well documented in the literature^5^^–^^7^ and appears to be the most frequently reported severe complication. We present a case of life-threatening haemorrhage as a consequence of a giant rectal faecaloma, another possible complication that has rarely been reported in the literature. Written informed consent was obtained from the patient’s relatives for publication of this case report, including the accompanying images.

## Clinical presentation

A 66-year-old male patient was brought in by ambulance to the accident and emergency department owing to reported profuse per-rectum (PR) haemorrhage. He was a nursing home resident with a medical history of advanced Parkinson’s disease and schizophrenia, with very limited communication and mobility. He was being artificially fed via a percutaneous gastrostomy tube. A history of chronic constipation was given by the nursing home staff who also reported a blood loss of approximately 1 l.

The patient presented with haemodynamic shock. On inspection, he was cachectic, very pale and drowsy. On examination, the Glasgow Coma Scale score was 11/15. He had cold extremities, with significant hypotension, tachycardia and tachypnoea. The initial recorded blood pressure (BP) was 67/46 mmHg, the pulse rate 94 beats min^–1^ and the respiratory rate 26 min^–1^. Laboratory blood tests revealed a haemoglobin concentration of 90 g l^−1^ and a platelet count of 153 × 10^9^l^–1^ with deranged renal function: serum creatinine of 177 mol l^–1^ and urea of 41.6 mmol l^–1^. There were no previous results available for comparison.

Initial resuscitation measures were implemented, including immediate transfusion of 1 unit of O negative packed red cells. Clinical examination confirmed ongoing PR bleeding and a large, hard central abdominal mass.

## Differential diagnosis

The PR discharge consisted mostly of fresh red blood rather than melaena, suggesting a lower gastrointestinal haemorrhage. Given the palpable abdominal mass, a large colonic tumour was suspected, while other possible aetiologies such as diverticular disease, inflammatory bowel disease, arteriovenous malformations or benign anorectal diseases (e.g. haemorrhoids) were believed to be less likely. However, given the amount of blood loss, the possibility of a fistula between the colon and an abdominal aortic aneurysm was raised. Following discussion with the on-call gastroenterology team, an urgent CT angiogram was requested.

## Imaging findings

**Figure 1.**
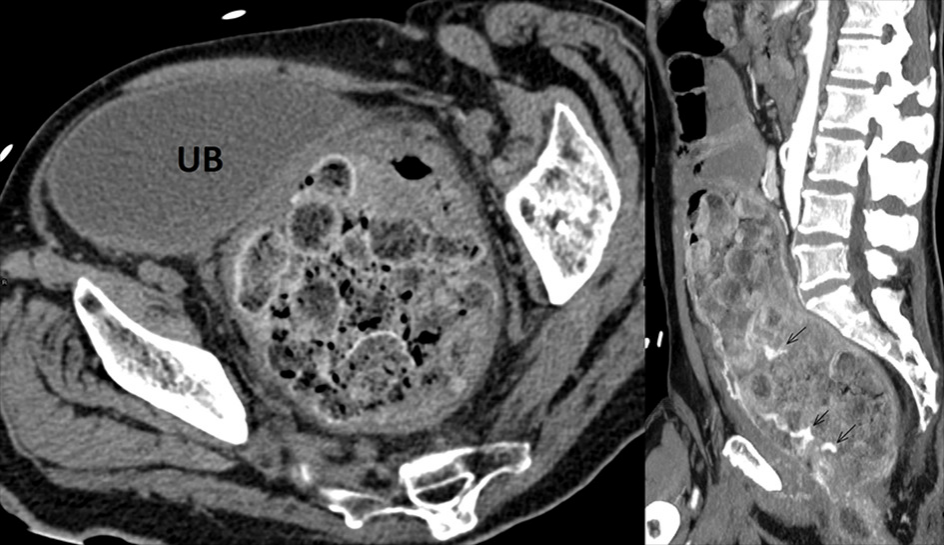
On the left, the axial unenhanced CT image shows the dilated rectum filled with numerous stercoral stones and causing extrinsic compression on the UB. On the right, a sagittal maximum intensity projection reconstruction of the arterial enhanced scan shows the true extent of the faecaloma and the intraluminal contrast material between the stercoral stones (arrows). UB, urinary bladder.

**Figure 2.**
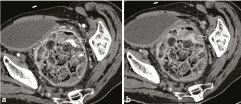
Axial CT images showing the large rectal faecaloma and intraluminal high-density material (arrows) consistent with contrast extravasation seen in the early arterial phase (a) and to a lesser extent on the delayed venous phase (b).

An urgent abdominal CT was performed using a triple-phase scan consisting of unenhanced arterial and portal venous phases. The unenhanced scan demonstrated a conglomerate of stercoral stones forming a large intraluminal rectal mass that measured approximately 12 × 12 × 25 cm (transverse and craniocaudal diameters) and occupied most of the abdominopelvic cavity (Figure 1). Significant mass effect was noted with compression of the urinary bladder, leading to moderate bilateral ureterohydronephrosis and stretching of the surrounding vasculature. The contrast-enhanced scans demonstrated accumulation of intraluminal contrast, visible in both arterial and portal venous phases but most prominent on the former, in keeping with active arterial haemorrhage from what seemed to be a branch of the left internal iliac artery (Figure 2).

## Treatment

Despite aggressive fluid resuscitation, repeated blood transfusions and administration of intravenous fresh frozen plasma and tranexamic acid, the PR haemorrhage persisted and the patient remained haemodynamically unstable with a recorded BP of 47/27 mmHg. The anaesthesia team was involved for inotropic support and general anaesthesia. Following discussion with the interventional radiologists, the patient was transferred to the angiography suite for embolization.

**Figure 3.**
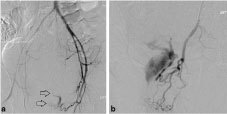
(a) Subtracted pelvic arteriogram image demonstrating obvious contrast extravasation on the initial run (arrows). (b) The extent of the haemorrhage is better seen following selective catheterization of the feeding vessel, a branch of the left internal iliac artery.

**Figure 4.**
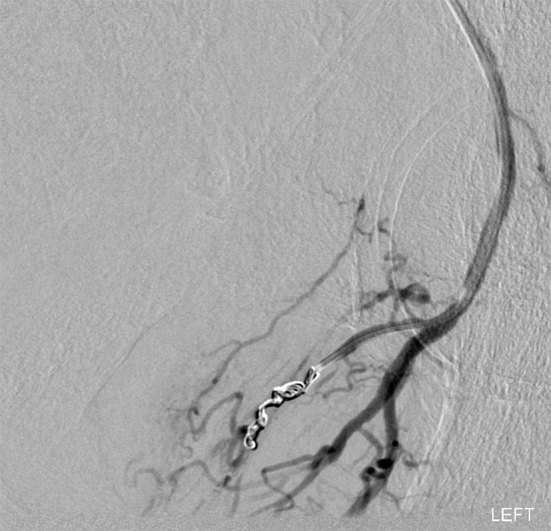
Angiographic image following coil embolization demonstrating a good outcome.

Vascular access was via an ultrasound-guided retrograde puncture in the right common femoral artery. An initial run of the left iliac system showed gross active extravasation from a branch of the internal iliac artery into the rectum, above the anal canal (Figure 3). The feeding vessel was selectively catheterized and embolized with 2- and 3-mm coils (Figure 4). Following this, angiography of the left inferior mesenteric artery and right internal iliac artery showed no evidence of extravasation from these vessels. Once control of the haemorrhage was achieved, there was rapid haemodynamic improvement with systolic BP values of 120–130 mmHg, compared with the pre-procedural values of 47–90 mmHg.

## Outcome and follow-up

The patient spent 4 days on the intensive therapy unit where he made a good recovery without major complications. PR discharge of small and decreasing amounts of fresh blood continued for the first 12 h following embolization but no further episodes of bleeding were noted after this time. In total, the patient was transfused 18 units of packed red cells. He was started on a regimen of laxatives and had numerous frequent bowel movements over the course of his admission. He was discharged from hospital 1 week after admission with no routine follow-up. He presented 12 months later at a different hospital with unrelated symptoms.

## Discussion

Chronic constipation is a common disorder with an average prevalence of 15% in the general population and increased frequency with increasing age.^8^ It has a negative impact on the quality of life and is a huge economic burden.^9^ Recent data show that it is associated with poorer survival.^10^ Given the ever-increasing life expectancy, a rise in the prevalence of chronic constipation is anticipated^9^ and both clinicians and radiologists should have knowledge of the possible complications. Faecal impaction and formation of faecalomas, in particular, can cause significant morbidity and mortality owing to large bowel obstruction or stercoral colitis and perforation, which are associated with poor prognosis. This is especially true since cases such as this are typically seen in elderly, frail patients with numerous comorbidities and little physiological reserve. Particularly institutionalized patients, nursing home residents or those with physical and mental impairment are at increased risk.^5^^–^ ^7,11^ A paper by Rey et al^11^ looking at residents from 34 nursing homes found a prevalence of over 70% and 47% for chronic constipation and faecal impaction, respectively. It is therefore in such a population that every effort should be directed towards prophylaxis to prevent faecaloma formation and its complications.

When a case such as this does present, the different treatment options available must be considered. In general, endoscopy would be the first line of investigation and/or treatment for severe lower gastrointestinal bleeding (GIB) in a haemodynamically stable patient. However, as opposed to cases of upper GIB, treatment options are limited and rarely provide a definitive solution. Endoscopy is therefore most often used as a diagnostic tool to identify the site and nature of the problem (e.g. rectal tumour *vs* sigmoid diverticulosis *vs* right colon angiodysplasia) and help guide the definitive treatment. Furthermore, most patients with lower GIB have a relatively empty bowel owing to frequent defecation induced by the haemorrhage. This case, however, is completely different, and whenever the lumen is impacted with solid faecal matter, insufflation or endoscopic visualization of the mucosa will not be possible.

In a case of less severe haemorrhage, rectal disimpaction could be considered to evacuate the bowel and allow endoscopic treatment. Laxative treatment would likely take too long to play a significant role in any patient with GIB and numerous cases of large faecalomas are likely to be resistant to laxative treatment and require surgical evacuation. Unfortunately, non-invasive methods have a limited role in clearing the rectum. Manual disimpaction, even aided by an anal retractor, is limited by a short reach. Oral purgatives, enemas or anorectal irrigation would be the remaining options, but none of these would be suitable in a patient with acute haemodynamic compromise from PR bleeding. Abdominal laparotomy to evacuate large faecalomas is a solution reported in the literature^1,2^ but carries the risks of any major surgical intervention.

In a patient with severe haemorrhage, a rapid definitive treatment would be required. Transanal surgery would have a very limited role in such patients and probably would not be considered unless the site of haemorrhage was known to be very close to the anal verge (i.e. 5–6 cm). In fact, not knowing the site of haemorrhage is one of the major issues with surgery. CT angiography may not always demonstrate the exact site of bleeding, and in the absence of such guidance, the standard procedure would be a subtotal colectomy with end ileostomy and closure of the rectal stump, which would leave the rectum in place. In the case presented in this paper, the rectum was the source of bleeding, meaning a high risk of continuous haemorrhage requiring a second laparotomy and a low anterior resection. As an emergency, such a procedure would have a very serious mortality risk and leave the patient with a permanent stoma.

Radiology therefore plays a decisive role in diagnosing and guiding the treatment of this life-threatening complication. In fact, several papers in the literature^5,6^ describe the essential role of prompt CT imaging in diagnosing other complications of faecal impaction such as stercoral colitis, which can lead to fatal perforation and peritonitis. In the case presented here, given the patient’s poor overall functional status and physiological reserves, it is believed that an open surgical intervention would have been fatal. Endoscopic treatment also would have been impossible given the degree of faecal impaction. Emergency endovascular treatment was undoubtedly the only treatment with significant chances of success and this case emphasizes the importance of having access to an interventional radiology department capable of promptly dealing with such presentations.

This case report is of a very unusual complication in a case of extreme coprostasis leading to giant faecaloma formation. At the same time, this is a typical case of faecal impaction where the patient had most of the risk factors discussed above. In addition, he presented with severe dehydration, as demonstrated by the very high urea levels, another recognized risk factor for faecal impaction. An abdominal mass in such a patient should raise the suspicion of faecaloma and prompt an urgent investigation with CT, which can easily offer an early diagnosis and possibly prevent fatal complications.

## Learning points

1. Faecal impaction is very common in debilitated elderly patients who are nursing home residents.
2. Efforts should be made to prevent faecaloma formation as the complications can be life-threatening and very difficult to treat.
3. In cases of large faecaloma formation, early investigation with CT can offer a prompt diagnosis and may prevent, or guide treatment of, serious complications.
4. Life-threatening haemorrhage is a rare but possible complication of large faecalomas and access to acute interventional radiology services can be life-saving.
